# The Inclusion of *Prosopis laevigata* Pods in Finishing Lamb Diets Affects Performance and Induces Non-Target Metabolomic Modifications in the Liver and Meat

**DOI:** 10.3390/ani16040666

**Published:** 2026-02-19

**Authors:** Héctor Aarón Lee-Rangel, Julio Alberto Navidad Maldonado, Rogelio Flores-Ramírez, Anayeli Vazquez-Valladolid, César Ilizarriturri-Hernandez, Oziel Montañez-Valdez, Alfonso Juventino Chay-Canul, Ana Karen Frutis-Moto

**Affiliations:** 1 Facultad de Agronomía y Veterinaria, Centro de Biociencias, Universidad Autónoma de San Luis Potosí, San Luis Potosí 78321, Mexico; julio_navidad30@outlook.com (J.A.N.M.); anayeli.vazquez@uaslp.mx (A.V.-V.); a348507@alumnos.uaslp.mx (A.K.F.-M.); 2 Coordinación para la Innovación y Aplicación de la Ciencia y la Tecnología—CIACYT, Universidad Autónoma de San Luis Potosí, Sierra Leona 550, San Luis Potosí 78120, Mexico; rogelio.flores@uaslp.mx (R.F.-R.); cesar.ilizaliturri@uaslp.mx (C.I.-H.); 3 Grupo de Investigación en Nutrición Animal (GINA), Centro Universitario del Sur, Universidad de Guadalajara, Ciudad Guzmán 49000, Mexico; oziel.montanez@cusur.udg.mx; 4 División Académica de Ciencias Agropecuarias, Universidad Juárez Autónoma de Tabasco, Carr. Villahermosa-Teapa, km 25, Villahermosa 86280, Mexico; aljuch@hotmail.com

**Keywords:** sheep, finishing, productivity, metabolomic, tannins

## Abstract

This study assessed the impact of the dietary inclusion of *Prosopis laevigata* pods (PLPs) on growth performance, carcass traits, and the metabolomic profiles of liver and meat in finishing lambs. A total of 28 crossbred lambs (38 ± 5 kg body weight) were allocated to one of two treatments: a control diet (0 g PLP/kg dry matter, n = 14; CONT) and a diet supplemented with 300 g PLP/kg dry matter (DM) (n = 14; PS). Growth performance was monitored over 25 days. Animals were assigned to a randomized design, and data were analyzed using the General Linear Model (GLM) procedure. Compared with the control diet, PLP inclusion (300 g/kg DM) reduced total body weight gain (*p* = 0.04) and worsened feed conversion efficiency. Lambs on the control diet also displayed a significantly greater (*p* = 0.02) rump perimeter. In contrast, lambs fed the 300 g PLP/kg DM diet showed a marked increase (*p* < 0.05) in the *longissimus thoracis et lumborum* (LTL) muscle area. Principal component analysis revealed a distinct separation between treatment groups based on the identified metabolites. Liver metabolomic data accounted for 30.6% of the total variability, while meat samples accounted for 45.7%. A total of 21 and 23 metabolites exhibited positive correlations in liver and meat, respectively. Notably, PLP supplementation influenced several metabolic pathways (*p* < 0.05), including the biosynthesis of unsaturated fatty acids, fatty acid biosynthesis, and sulfur metabolism in both liver and meat. Additionally, phenylalanine metabolism was specifically affected (*p* < 0.05) in the liver, while steroid biosynthesis was altered (*p* < 0.05) in meat. Overall, the inclusion of PLPs in the diet of finishing lambs resulted in notable changes to the liver and meat metabolomes, particularly in pathways associated with fatty acid biosynthesis. Although PLP supplementation reduced overall growth performance, it did not negatively impact carcass quality traits; hence, we recommend the inclusion of 300 g PLP/kg DM in finishing lamb diets.

## Simple Summary

*Prosopis laevigata* pods are a locally available feed resource that could help reduce feeding costs in lamb production, but their effects on animal performance and meat characteristics need to be clarified. This study evaluated the inclusion of *Prosopis laevigata* pods in diets for finishing lambs and their effects on growth, carcass traits, and chemical changes in the liver and meat. Twenty-eight lambs were fed either a control diet or a diet containing *Prosopis laevigata* pods for 25 days. Lambs receiving the supplemented diet showed lower body weight gain and poorer feed conversion efficiency compared with lambs fed the control diet. However, carcass quality was not negatively affected, and lambs fed *Prosopis laevigata* pods showed an increase in loin muscle area, which is an important trait for meat yield. In addition, the supplemented diet caused clear changes in the chemical composition of liver and meat, mainly related to fat synthesis and fat-related processes. The results suggest that *Prosopis laevigata* pods can be included in finishing lamb diets as an alternative feed ingredient without compromising carcass traits, supporting the use of local resources in animal production.

## 1. Introduction

Small ruminants have relevant socio-economic and ecological value, particularly in low- and middle-income countries where they support household nutrition and income [1,2]. In Mexico, the availability of native pastures has promoted sheep production in certain regions, although the sector continues to face supply chain constraints and growing consumer concerns regarding the environmental, health, and welfare implications of intensive production systems [3,4].

In recent years, the growing demand for lamb meat in Mexico has increased reliance on intensive lamb finishing systems [5]. According to the Mexican Ministry of Agriculture and Rural Development (SADER), per capita lamb meat consumption increased from 0.6 kg/person in 2020 to 1.0 kg/person in 2024. These finishing systems commonly use energy-dense, grain-based diets to meet the high nutritional requirements of lambs during the final growth phase [6]. Nevertheless, the high cost of cereal grains and their frequent inclusion at levels exceeding 60% of the total diet can compromise profitability and increase the risk of ruminal acidosis, negatively affecting feed intake and average daily gain [7].

To improve the economic viability of lamb production, several alternative feed resources have been evaluated as partial substitutes for cereal grains, including agro-industrial by-products (e.g., dried citrus pulp, distillers dried grains with solubles, and olive cake), crop residues, and other non-conventional plant resources, with variable effects on growth performance and carcass traits [8,9,10].

Mesquite (*Prosopis* spp.), a leguminous tree widely adapted to arid and semi-arid regions of Mexico, South America, and the Caribbean, has received particular attention. Its pods represent a locally available feed resource and contain approximately 329 g/kg neutral detergent fiber (NDF), 78 g/kg crude protein (CP), 21 g/kg ether extract (EE), and 250 mg/kg tannins on a dry matter (DM) basis [11].

Previous studies evaluating mesquite pod inclusion in small ruminant diets have reported that supplementation up to 300 g/kg DM does not compromise growth performance in sheep and goats. Cook et al. [12] reported no evidence of toxicity in goats during short-term mesquite pod consumption; however, higher inclusion levels (600 g/kg DM) were associated with reduced weight gain, likely due to the presence of anti-nutritional compounds. Similarly, Negrete et al. [13] and Peña-Avelino et al. [11] showed that mesquite pods can partially replace conventional grains at inclusion levels of 300–500 g/kg DM, reducing feeding costs without adversely affecting animal health or productivity. In particular, these authors reported reductions in diet cost of up to 21% when *Prosopis laevigata* pods (PLPs) were included.

More recently, metabolomics has emerged as a powerful tool for studying muscle biology and meat quality, providing detailed insights into metabolic changes associated with nutritional interventions [14,15]. This approach has been increasingly applied in animal science to evaluate the physiological effects of feeding strategies and their impact on traits such as growth, fat deposition, and meat quality [16]. We hypothesized that supplementing finishing lamb diets with *Prosopis laevigata* pods (PLPs) could influence productive traits by modulating key metabolic pathways in the liver and muscle. Therefore, this study aimed to evaluate the effects of PLP inclusion on growth performance, carcass characteristics, and the non-target metabolomic profiles of liver and meat tissues in lambs under an intensive finishing regime.

## 2. Materials and Methods

The trial was conducted at the Small Ruminant Production Unit, Faculty of Agronomy and Veterinary Medicine, Autonomous University of San Luis Potosí (UASLP), Mexico (φ 22°14′0.58; λ 100°50′48.5), during the spring–summer of 2024. The experimental procedures were submitted for review and approved by the Institutional committee for the care and use of experimental animals of the Faculty of Agronomy and Veterinary (protocol ID: IAZ-2023-002).

### 2.1. Animals, Housing, and Experimental Design

Twenty-eight male Rambouillet × Suffolk crossbred lambs (initial BW 38.07 ± 4.68 kg, four months age) were individually housed in metabolic crates (0.80 m × 1.20 m) with individual feeders and drinkers, following the guidelines established by the Mexican Government’s federal laws in NOM-051-ZOO-1993 [17], NOM-033-ZOO-1995 [18], and NOM-062-ZOO-1999 [19]. Before the trial, animals were dewormed and supplemented with vitamins A, D, and E.

Lambs were randomly assigned to one of two dietary treatments (n = 14): (1) a control diet (CONT), and (2) a diet that contained ground *P. laevigata* pods (PS). Both diets were formulated to meet NRC [20] (Table 1) requirements and were isoenergetic and isonitrogenous. *P. laevigata* pods were included by partially substituting corn, wheat bran, and oat hay. Feed was offered twice daily (07:00 and 16:00 h), ensuring 10% refusals to allow for ad libitum intake. Composite feed samples were collected weekly and analyzed for proximate composition [21], NDF [22], and condensed tannins [23].

The finishing period lasted 25 days following a 10-day adaptation phase (increasing 10% daily). Lambs were weighed at the beginning and end of the trial to calculate average daily gain (ADG), and feed conversion efficiency (FCE) was computed from feed intake and weight gain.

### 2.2. Slaughter Procedures and Carcass Evaluation

Slaughter was performed at a commercial abattoir according to NOM-08-ZOO [24] (stablish the requirements for the establishments for the slaughter and processing of meat products), NOM-09-ZOO [25] (refers to sanitary procedures for the slaughter and the processing of their products of domestic animals), and NOM-033-ZOO [18] standards (refers to the humane slaughter of domestic and wild animals). Hot carcass weights were recorded immediately post-slaughter. After 24 h of chilling at 4 °C, *longissimus thoracis et lumborum* (LTL) samples (100 g) were collected and frozen (−20 °C) for later analysis. Carcass traits were assessed following Colomer-Rocher [26], including ribeye area and the weight of non-carcass components (e.g., head, organs, skin, feet). Liver samples (50 g) were also collected and stored at −20 °C.

### 2.3. GC–MS Metabolite Profiling

Liver and muscle samples (1 g) were homogenized with 10 mL of hexane/acetone (75:25, *v*/*v*) using an ultrasonic processor (GEX130, Cole-Parmer, Vernon Hills, IL, USA). The organic phase was concentrated to 1 mL using a Zymark Turbovap LV evaporator (Zymark, Hopkinton, MA, USA). Extracts were analyzed by using gas chromatography coupled to mass spectrometry (GC–MS; HP 6890 GC with HP 5973 MS, Agilent Technologies, Santa Clara, CA, USA) equipped with an HP-5MS capillary column (60 m × 0.25 mm × 0.25 µm). The oven temperature program was set from 70 to 310 °C using programmed ramps. Helium was used as carrier gas at 1 mL/min, and the injector was operated in splitless mode at 250 °C. Mass spectra were acquired in full-scan mode (*m*/*z* 50–500). Metabolites were tentatively identified by mass spectral library matching and supported by Kovats retention indices (RIs). Only compounds with an identification quality score ≥ 80% were retained for downstream statistical analysis.

### 2.4. Statistical Analysis

Performance and carcass data were analyzed using the GLM procedure in SAS 9.0 [27], and treatment means were compared using Tukey’s test (*p* ≤ 0.05); the initial body weight was used as a covariate. Metabolomic data were processed using MetaboAnalyst 6.0, with log transformation and normalization applied before multivariate analysis. Orthogonal partial least squares discriminant analysis (OPLS-DA) was performed using MetaboAnalyst 6.0 to identify discriminant metabolites between experimental groups. The OPLS-DA model separates predictive variation related to class discrimination from orthogonal variation unrelated to the response variable. Model performance was assessed using R^2^X, R^2^Y, and Q^2^ metrics, and model validity was confirmed via permutation testing. Discriminant metabolites were selected based on variable importance in projection (VIP) scores derived from the predictive component (VIP > 1.0) in combination with loading correlation values (p(corr)) and univariate statistical significance (*p* < 0.05), and metabolic pathways were identified using the KEGG database.

## 3. Results

### 3.1. Productive Performance

Compared with the control diet, PLP supplementation reduced total weight gain and feed conversion efficiency (*p* < 0.05). Initial body weight, final body weight, dry matter intake, and feed conversion ratio did not differ between treatments. Average daily gain tended to be higher in PLP-fed lambs (*p* = 0.09) (Table 2).

### 3.2. Carcass Characteristics

Compared with the control diet (CONT), PLP supplementation reduced rump perimeter (*p* < 0.05) and increased the *longissimus thoracis et lumborum* (LTL) area (*p* < 0.05). Hot carcass weight, carcass length, leg length, rump depth, thorax perimeter, thorax depth, dorsal fat, and carcass yield did not differ between treatments (Table 3).

### 3.3. Non-Target Metabolomic Study of Liver and Meat

**Figure 1:**
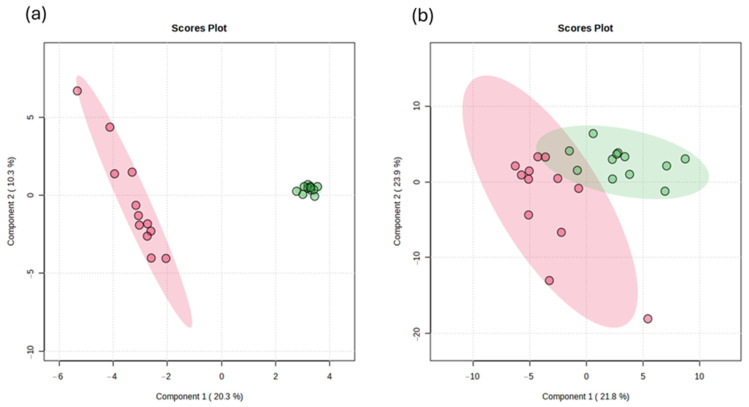
Orthogonal partial least squares discriminant analysis of liver (**a**) and meat (**b**) compounds from finishing lambs fed with *P. laevigata* pods.

Orthogonal partial least squares discriminant analysis (OPLS-DA) was performed to analyze the metabolites identified in liver and meat tissues. In liver tissue, the OPLS-DA model showed a clear separation between treatments. The optimal model (two components) achieved an accuracy of 1.00, with high explained variance (R^2^ = 0.982) and strong predictive ability (Q^2^ = 0.929), indicating a robust classification performance (Figure 1a). In meat tissue, OPLS-DA also discriminated between treatments. The optimal model (four components) showed an accuracy of 0.837, with R^2^ = 0.971 and Q^2^ = 0.694, suggesting a satisfactory model fit and predictive capacity (Figure 1b). The model was validated with 1000 permutations (*p* < 0.05), confirming that the metabolic profiles of compounds in both liver and meat tissue were significantly altered.

**Figure 2:**
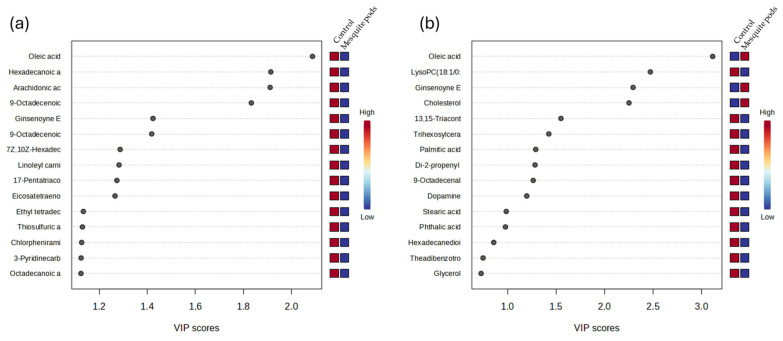
Variable importance in projection (VIP) plot for liver (**a**) and meat (**b**) compounds from finishing lambs fed with *P. laevigata* pods.

A total of 56 and 46 metabolites were identified in liver and meat tissues, respectively. The top 15 metabolites distinguishing liver and meat tissues are presented in Figure 2. The metabolites in liver tissue were predominantly associated with the CONT treatment. According to the variable importance in projection (VIP) scores, all 15 major compounds in liver tissue were linked to the CONT diet. Conversely, in meat tissue, oleic acid, lysophosphatidylcholine (LysoPC), ginsenoyne, and cholesterol were more strongly associated with the PLP treatment (VIP score > 2; Figure 2).

**Figure 3:**
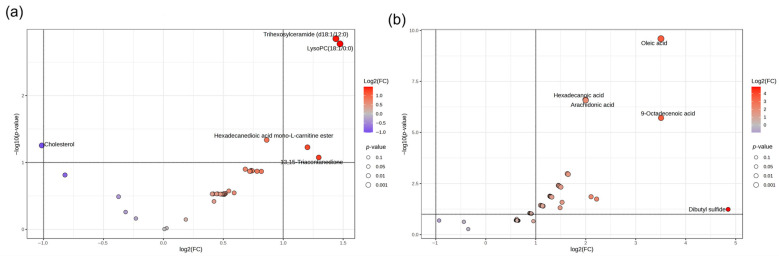
Volcano plot of liver (**a**) and meat (**b**) compounds from finishing lambs fed with mesquite pods.

The volcano plot analysis revealed that trihexosylceramide, lysophosphatidylcholine (LPC), L-carnitine ester, hexadecanoic acid, and triacontanedione were upregulated (fold change ≥ 1, *p* < 0.05) in liver tissue, whereas cholesterol was downregulated (fold change ≤ 1, *p* < 0.05; Figure 3a). In meat tissue, oleic acid, hexadecanoic acid, arachidonic acid, 9-octadecanoic acid, and dibutyl sulfide were upregulated (fold change ≥ 1, *p* < 0.05), with no downregulated compounds observed (Figure 3b).

**Figure 4:**
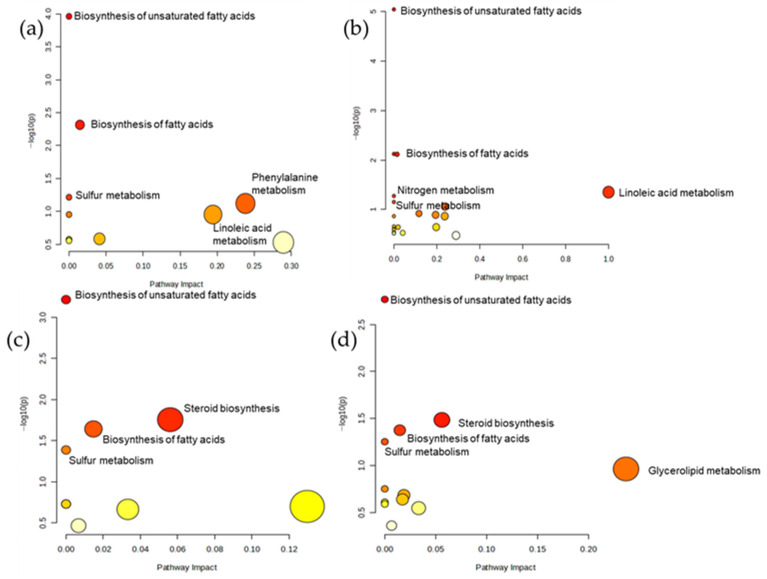
Liver ((**a**) [CONT] and (**b**) [PLP]) and meat ((**c**) [CONT] and (**d**) [PLP]) metabolomics analysis according to the KEGG pathway database.

Figure 4 further illustrates the specific metabolic pathways influenced by the CONT and PLP diets. In liver tissue (Figure 4a,b), the PLP diet upregulated pathways related to unsaturated fatty acid biosynthesis, fatty acid biosynthesis, and sulfur metabolism compared with the CONT diet. Similarly, in meat tissue (Figure 4c,d), the PLP diet induced upregulation in pathways associated with the biosynthesis of unsaturated fatty acids, sulfur metabolism, and steroid biosynthesis.

## 4. Discussion

In agreement with previous reports evaluating *Prosopis* pods in small ruminant diets [11,13], the inclusion of PLPs in our study did not negatively affect feed intake, indicating acceptable palatability and intake regulation under the finishing conditions used. This is relevant from a practical standpoint, since finishing systems commonly rely on highly fermentable cereal-based diets, and alternative ingredients must maintain intake to sustain growth.

Despite the similar intake, total weight gain and feed conversion efficiency were higher in lambs fed the control diet. This suggests that nutrient utilization was more favorable in the control group, potentially due to differences in ruminal degradation dynamics and nutrient availability. A plausible explanation involves the presence of condensed tannins in PLPs. Tannins can exert beneficial effects at low-to-moderate inclusion rates by reducing excessive ruminal protein degradation, improving protein–energy synchronization, and enhancing microbial protein synthesis [28]. However, depending on dose and chemical structure, tannins may also form complexes with dietary protein and carbohydrates, reducing apparent digestibility and ruminal degradability [29]. Under such circumstances, nutrient availability for growth may decline even when intake remains unchanged, which could partly explain the lower total weight gain and reduced efficiency observed in PLP-fed lambs. The tannin concentration in our diet (9.4%) may have been low enough to promote the formation of complexes between carbohydrates and proteins, potentially reducing the apparent digestibility and rumen degradability of dry matter, organic matter, and crude protein.

Some studies report non-linear responses to tannin supplementation, where moderate doses do not impair performance, but higher effective concentrations or more reactive tannin fractions may negatively affect utilization [30,31]. In our diet, the tannin content from PLPs may have been sufficient to influence ruminal digestion kinetics, affecting passage rate and potentially increasing rumen fill over time [29], ultimately reducing efficiency.

Most carcass variables, including hot carcass weight, dressing percentage, carcass length, dorsal fat, and thoracic measurements, were not affected by PLP supplementation. These results support the concept that external carcass measurements are relatively stable and are not easily modified by dietary interventions unless they substantially modify energy flux and tissue deposition patterns [32,33]. Thus, under the present finishing conditions, PLPs maintained carcass development and commercial yield.

However, specific differences were detected: the rump perimeter was higher in the control treatment, whereas the area of the *longissimus thoracis et lumborum* (LTL) was greater in PLP-fed lambs. The latter finding is particularly relevant because LTL area is a sensitive indicator of muscularity and may reflect shifts in nutrient partitioning [34]. Although our experiment did not include detailed carcass composition analyses, the larger LTL area in PLP-fed lambs suggests that PLPs may modulate muscle deposition in targeted anatomical regions. Similar variability in carcass responses to mesquite pods has been documented across studies, largely dependent on inclusion rate, animal category, and basal diet [11,13,35]. Together, the present carcass results indicate that PLPs can be used without impairing yield, while potential improvements in selected muscle traits merit further evaluation with more detailed carcass dissection and meat quality assessment.

A major contribution of the present work is the integration of non-target metabolomics to evaluate how PLP supplementation affects metabolism in liver and muscle. The clear separation between treatments observed in OPLS-DA, together with permutation validation, confirms that PLPs induced robust metabolic changes in both tissues. This supports the view that metabolomics is a valuable approach to uncover biological effects of non-conventional feed ingredients beyond performance indicators [14,15,16].

In liver tissue, the volcano plot results indicate diet-associated differential abundance of compounds such as lysophosphatidylcholine (LPC) and cholesterol. LPC is generated from phosphatidylcholine through enzymatic mechanisms involving phospholipase activity and remodeling processes, and it plays a role in lipid signaling and cellular homeostasis [36,37]. Altered hepatic LPC abundance may reflect modifications in membrane lipid turnover, lipoprotein metabolism, or oxidative status [38].

The observed reduction in cholesterol in PLP-fed animals may indicate diet-induced shifts in sterol metabolism. Such changes could be mediated by altered nutrient absorption, modifications in hepatic lipid handling, or changes in ruminal biohydrogenation and subsequent lipid supply to the liver [39]. Although mechanistic interpretation must be cautious, these results suggest that PLP affects hepatic lipid regulation, which is consistent with the pathway enrichment indicating responses involving fatty acid and sterol-related metabolism.

In meat tissue, several discriminant metabolites with high VIP scores were associated with PLPs, including oleic acid and cholesterol-related signals, with a general upregulation of lipid-related compounds (e.g., oleic acid, arachidonic acid, hexadecanoic acid). These metabolites are directly relevant to meat lipid composition and could influence the eating quality, nutritional value, and oxidative stability. Oleic acid is often associated with improved sensory acceptability and healthier fatty acid profiles, while arachidonic acid is a biologically active fatty acid involved in inflammatory signaling and membrane dynamics [40].

The pathway analysis highlighted enrichment of unsaturated fatty acid biosynthesis, sulfur metabolism, and steroid biosynthesis in PLP-fed lambs in both liver and meat tissues. Collectively, these findings suggest that PLP inclusion may influence ruminal lipid transformations and systemic lipid handling. Condensed tannins can inhibit specific rumen microbial populations involved in biohydrogenation, potentially decreasing the saturation of dietary polyunsaturated fatty acids and increasing the availability of unsaturated fatty acids for absorption and deposition [39]. This provides a plausible nutritional mechanism linking PLP composition with the metabolomic signatures observed in muscle.

Beyond rumen-driven mechanisms, diet-induced lipid shifts could also involve differences in tissue regulation of lipid synthesis and storage. Transcription factors such as PPARγ play key roles in lipid uptake and adipogenesis and may be influenced by dietary components and energy balance [41]. Although gene expression was not evaluated in our study, the combined metabolomic patterns support the hypothesis that PLPs modulate lipid-related metabolic processes at systemic and tissue levels.

## 5. Conclusions

The inclusion of *Prosopis laevigata* pods (PLPs) in finishing lamb diets during a 25-day feeding period affected productive performance, as lambs fed the control diet showed higher total weight gain and better feed conversion efficiency than those supplemented with PLPs. Most carcass traits and carcass yield were not influenced by PLPs; however, rump perimeter was greater in the control group, whereas the *longissimus thoracis et lumborum* (LTL) area increased in lambs receiving PLPs. Non-target metabolomic analysis revealed clear diet-dependent metabolic differentiation in both liver and meat tissues, with major changes mainly associated with lipid-related metabolites and pathways, particularly fatty acid biosynthesis and metabolism. PLPs can be considered a locally available alternative feed resource for lamb finishing diets as a partial substitute for conventional ingredients; however, inclusion level and diet formulation should be optimized to avoid reductions in growth efficiency. Further studies integrating digestibility measurements and targeted lipidomics are warranted to confirm the effects of PLPs on meat lipid composition and quality.

## Figures and Tables

**Table 1:** Experimental diets and chemical composition (% dry matter (DM) basis).

Ingredient, % of DM	Control	Mesquite Pods
Corn grain ground	18.4	15
Corn grain cracked	18.3	15
Corn grain whole	18.3	15
Soybean Meal	13	13
Wheat bran	15	5
Oat hay	15	5
Premix ^1^	2	2
Mesquite pods	-	30
Chemical composition, %		
Dry matter	89	90
Crude Protein	15.4	15.2
Neutral Detergent Fiber	37.2	39.8
Acid Detergent Fiber	15.1	16.0
Ether extract	3.5	4.1
Ash	6.2	7.6
Total Tannins	6.6	9.4

^1^ Biotecap: Ca 1.88%, P 2.20%, Mg 1.40%, Na 0.035%, Cl 0.15%, Se 18.65 ppm, Cr 5 ppm, Cu 500 ppm, Zn 2050 ppm, Mn 215 ppm, Co 13.50 ppm, I 23.80 ppm, Fe 5000 ppm. Live yeast 5% (*Saccharomyces cerevisiae* 2 × 1010 CFU/g).

**Table 2:** Productive performance of finishing lambs in a diet that contains 300 g/kg DM of *P. laevigata* pods.

Item	Control	Mesquite Pods	SEM	SD	*p*-Value
Initial Body Weight, kg	37.6	38.4	1.99	4.65	0.36
Final Body Weight, kg	44.2	43.6	2.12	7.93	0.65
Total Gain, kg	6.6 ^b^	5.2 ^a^	0.84	3.14	0.04
Average Daily Gain (ADG), kg	0.264	0.208	0.035	0.131	0.09
Dry Matter Intake (DMI), kg	1.60	1.68	0.109	0.408	0.12
Feed Conversion, DMI:ADG ratio	6.01 ^a^	8.07 ^b^	0.01	0.137	0.03

Means followed by different lower-case letters in the same row are significantly different (*p* < 0.05); SEM, standard error of the mean; SD, standart desviation.

**Table 3:** Carcass characteristics of finishing lambs in a diet that contains 300 g/kg DM of *P. laevigata* pods.

Item	Control	Mesquite Pods	SEM	SD	*p*-Value
Hot carcass weight, kg	20.3	20.1	1.65	6.17	1.48
Carcass length, cm	56	59	1.38	5.16	3.86
Leg length, cm	45.2	44.2	0.9	3.37	1.47
Rump perimeter, cm	58 ^a^	50 ^b^	3.6	13.37	0.02
Rump depth, cm	25	23.7	1.38	5.16	2.82
Torax perimeter, cm	76	78.5	2.15	8.04	1.15
Torax depth, cm	42.5	43	2.65	9.92	3.24
Dorsal fat, cm	0.1	0.1	0.001	0.003	0.18
Area *longissimus thoracis et lumborum*, cm	24.8 ^a^	34.6 ^b^	3.85	14.41	0.05
Carcass yield, %	46	46.2	0.83	3.10	1.70

Means followed by different lower-case letters in the same row are significantly different (*p* < 0.05); SEM, standard error of the mean; SD, standart desviation.

## Data Availability

The data presented in this study are available on request from the corresponding author as they are contained in an institutional repository from a Basic in Science Thesis.
